# Mechanophilia – a case report of a specific fetishistic disorder

**DOI:** 10.3389/fpsyt.2026.1749802

**Published:** 2026-02-03

**Authors:** Peter Osvath, Franciska Könczöl, Sándor Fekete, Tamás Tényi

**Affiliations:** 1 Department of Psychiatry and Psychotherapy, Medical School, University of Pécs, Pécs, Hungary; 2 Institute of Forensic Medicine, Medical School, University of Pécs, Pécs, Hungary

**Keywords:** forensic psychiatry, mechanophilia, objectophilia, paraphilia, sexuality

## Abstract

In our case report, we present a very rare variant of fetishism, mechanophilia, by describing a case from our forensic psychiatric practice. Mechanophilia is a form of fetishism classified as a paraphilia, when a person is sexually aroused by various vehicles, most often cars, less often bicycles or airplanes. We report on the forensic psychiatric examination of a middle-aged man, who opened the fuel filler openings of cars parked in the yard of a car service, then smeared a substance of unknown origin into the tank and then performed a sexual act by placing his genitals in the filling openings. Our examination confirmed a sexual disorder, fetishism (including mechanophilia), which developed in addition to mild intellectual disability (IQ:66) - classified as a paraphilia.

## Introduction

Mechanophilia (also referred to as mechaphilia) is a rare form of fetishism classified among paraphilias, in which an individual experiences sexual arousal from vehicles—most commonly cars, and less frequently bicycles or airplanes (1–3). In fetishism, sexual desire typically requires an inanimate object, such as women’s underwear, stockings, shoes, or a non-genital body part, most often the female foot (4). The concept of fetishism was first described in the 19th century in the classic works of Krafft-Ebing (5) and Havelock Ellis (6). The term “fetishism” derives from the French fétiche and Portuguese feitico (meaning charm or talisman).

A mechanophilic individual experiences sexual excitement in relation to vehicles and may masturbate inside the vehicle, into the vehicle, or in its immediate vicinity. In psychiatric literature, only two cases have been reported with sufficient detail and scientific rigor (7, 8), whereas tabloid media has published multiple accounts that often prove unreliable (2).

In 1992, De Silva and Pernet (7) described the case of a 20-year-old British man characterized by marked social withdrawal and shyness. He had no sexual or romantic relationships and belonged, along with his family, to a strict religious sect. His behavior changed when his parents purchased an Austin Metro car. Initially, he began masturbating inside the vehicle, later performing autoerotic acts behind the car, near the exhaust pipe. For George, sexual arousal was triggered by the functioning exhaust and the emission of exhaust fumes. Prior to the onset of mechanophilic activity, his source of sexual excitement since the age of 10 had been voyeurism—watching others urinate—starting with dogs, then children, and eventually adult women. His only sexual experience occurred at age 14, when he was sexually abused by an older man.

In 1993, O’Halloran and Dietz (8) reported two cases in which men used tractor hydraulic arms for suspension during masochistic sexual stimulation. Tragically, both individuals died as a result of fatal accidents associated with these paraphilic practices.

In our study, we present the forensic psychiatric evaluation of a middle-aged man who forcibly opened the fuel filler caps of cars parked in a service yard, smeared an unknown substance into the tanks, and subsequently engaged in sexual acts by inserting his genitals into the filler openings.

## Case report

The subject, a middle-aged man (personal details anonymized), underwent forensic psychiatric evaluation in 2019, following charges of significant property damage. This took place four months after the incident. The examination was performed by two forensic psychiatrists and one forensic medical specialist (the first, second, and third author of the case report). During the assessment, he cooperated only partially. He reported living with his elderly mother, having no employment or independent income, and regularly using drugs (primarily synthetic cannabinoids), benzodiazepines, and large amounts of alcohol. At the time of examination, he complained of vague psychological symptoms, irritability, and episodes of aggression; his mood was labile, often tense and depressive. According to his statement, he was not taking any psychotropic drugs at the time of the investigation or the incident.

During the examination, the patient said: “I don’t know what day it is, and I don’t care, I know it’s September and 2019… I’m here because the police brought me in … but I don’t feel like talking about it, leave me alone … there was that car thing when they caught me with the car….

I often think about cars, about doing this with cars … When this desire comes over me and I see a car, I immediately get excited. The color or model doesn’t matter, the only thing that matters is that I can live out what comes over me at times like this….

Most of the time I get very tense because I know I have to control myself, otherwise I’ll get punished. But I don’t know why they’re asking me so many questions, leave me alone, it’s none of your business”.

Regarding the act, he stated that his urge to perform sexual acts with cars developed after a previous brain injury. Based on his medical record, there was no evidence of previous head injury, and the patient’s own interpretation did not seem clinically relevant. He had never been in a romantic relationship and frequently engaged in masturbation. His sexual attraction to cars had emerged years earlier because, as he explained, “cars are not as rejecting.” The color or type of vehicle did not matter. The patient experienced sexual fantasies with mechanophilic content numerous occasions without acting on them. When he felt sexual urges, he found them very difficult to control, though he usually resisted because he was aware of the legal consequences. It is important to clarify, that the subject did not consumed internet contents related to his mechanophilia.

Mental status examination – which was a two-hour-long interview done twice - showed partially adequate responses, partial orientation, slow comprehension, marked attention deficits, and impoverished thought processes. No psychotic content or perceptual disturbances were observed, and no suicidal intent emerged. His mood was labile, tense, irritable, occasionally restless and aggressive, with mildly impaired judgment and insight. Cognitive and intellectual functions were mildly deteriorated, with poor abstraction and reasoning abilities, though reality testing was largely preserved.

Psychological assessment (Rorschach Inkblot Test, Minnesota Multiphasic Personality Inventory (MMPI), Wechsler Adult Intelligence Scale (WAIS)) indicated low intellectual capacity, infantile traits, immature, primitive defense mechanisms, and poor impulse control. His behavior was dominated by impulse-control disorder, narcissistic tendencies, heightened sensitivity, aggressive fantasies, and low frustration tolerance. The psychological evaluation confirmed multiple abnormal personality traits, including deviant sexual behavior (infantile, polymorphous gratification, absence of interpersonal intimacy).

Based on these findings of psychiatric examination, a rare form of fetishism—mechanophilia—was diagnosed according to the diagnostic criteria of the DSM-5-TR (9).

His forensic history revealed prior proceedings in 2017, for theft involving property damage, during which he had also undergone psychiatric evaluation. At that time, he repeatedly removed fuel caps from various cars and smeared grease around the filler openings, although no sexual activity was confirmed. He claimed those acts were motivated by a desire to take revenge on neighbors who harassed him. Previous evaluations indicated intellectual performance at the lower limit of normal, but this did not impair his ability to understand the consequences of his actions.

Based on the medical record his psychiatric history revealed 15 years of continuous psychiatric care for “unspecified mental retardation with mild behavioral deterioration.” He had been treated with various psychotropic medications (chlorprothixene, alprazolam, biperiden) and received flupentixol depot injections, because of his poor compliance with the treatment. No significant somatic illnesses were reported.

His life history was marked by disharmonious personality development. Both parents were alcohol-dependent and died young. He grew up in foster care and recalled little of his childhood. He completed eight grades in a special elementary school but never acquired a profession. Due to his underlying mental disorder, he was declared disabled and placed under partial guardianship. In recent years, he worked intermittently in sheltered employment.

The forensic psychiatric evaluation concluded that the subject was neither incapable nor significantly impaired in recognizing the consequences of his actions or acting accordingly at the time of the offense or during the examination.

The information obtained during the follow-up period revealed that he had two psychiatric hospitalizations: in 2020, following a suicide attempt by hanging, he was treated for mild intellectual disability with marked behavioral deterioration, acute and transient psychotic disorder, and adjustment disorders, receiving haloperidol, clonazepam, quetiapine, later olanzapine, biperiden orally, and olanzapine depot injections. In 2021, he was hospitalized again due to homicidal and suicidal statements and aggressive behavior. During this admission, in addition to previous diagnoses, undifferentiated schizophrenia was established. The treatment regimen continued as previously prescribed. He regularly attends outpatient psychiatric care. With psychosocial rehabilitation as part of community psychiatric care and regular olanzapine depot injections, he didn’t have any productive psychotic symptoms, his impulsiveness decreased, and he was able to control his sexual impulses properly. He was able to take on rehabilitation work and did not commit any further paraphilic or other crimes.

## Discussion

In our case a rare form of fetishism—mechanophilia—was diagnosed according to the diagnostic criteria of the DSM-5-TR (9). In the International Classification of Diseases (ICD) version 10. fetishism is coded as F65.0 (within Paraphilias, falling under Disorders of Adult Personality and Behavior (F60-F69). It is worth noting that the separate code F65.0 for fetishism, which was previously included in ICD-10, has been removed from ICD-11. In the new ICD-11 classification system, sexual preference is only considered abnormal if the sexual impulse triggered by the object causes significant distress and functional impairment, and is not merely a matter of specific sexual preference. In this case a diagnosis of Paraphilic disorder involving solitary behavior or consenting individuals (6D36) should be used (10). In our case the diagnosis is based on the fact that our patient experienced recurrent and intense sexual arousal (manifested by fantasies, urges, and behavior) from the use of cars. This symptoms presented chronically, more years. He found it very difficult to control his sexual impulses, which caused him significant distress, and he was terrified of being noticed and punished.

In our case the comprehensive forensic evaluation confirmed a sexual disorder—fetishism (specifically mechanophilia)—associated with mild intellectual disability (IQ: 66). It is well-documented that certain paraphilias, such as pedophilia, are more prevalent among individuals with lower IQ (11), so our finding on the intellectual level of our patient can be considered as important. The patient had never been in a romantic relationship and had no prior sexual contact with either women or men. His mechanophilic behavior persisted for several years; although he attempted to control his urges due to fear of punishment, they periodically broke through, leading to repeated damage to vehicles and sexual acts involving them. During episodes of mechanophilic activity, no psychotic thought content or perceptual disturbances were observed, and reality testing remained intact. Psychological assessment revealed multiple abnormal personality traits, including deviant sexual behavior (infantile, polymorphous gratification, absence of interpersonal intimacy). From a psychodynamic perspective in the background of infantile, deviant sexual behavior, developmental problems and regression to primitive object relations can be assumed (4).

Although altered, paraphilic sexual behavior may be seen more frequently in patients with schizophrenia (12) and the follow-up of our patient later revealed psychotic symptoms; however, in our view, these were not etiopathogenetically related to the mechanophilic presentation described in this case. The paraphilia was already present before to the first symptoms of schizophrenia appeared. In addition to deficits in personality development, mild intellectual impairment, psychoactive substance use and associated impulse-control difficulties contributed to the persistence of paraphilic behavior. Fetishism may coexist with addiction as comorbidity (13). Another point to be mentioned is that symptoms of fetishism can increase and decrease with more and less substance administration, indicating that symptoms of paraphilia can emerge as a result of illicit drug use (13).

Mechanophilia—a form of fetishism classified among paraphilias—must be distinguished from objectophilia, a phenomenon that has attracted increasing attention in recent years (3, 14). Objectophilia (or Objectum Sexuality) is a sexual orientation disorder characterized by emotional, romantic, or sexual attraction toward specific objects (3, 14). Unlike mechanophilia, objectophilic individuals often do not engage in sexual acts with the objects they love or idolize. Two widely cited examples include Eija-Ritta Eklof Berliner-Mauer, who coined the term objectophilia and married the Berlin Wall in 1979, and Erika Eiffel, who committed herself to the Eiffel Tower in 2007 and adopted its name (3, 14). Objectophiles define themselves as a new sexual minority, creating online forums, societies, and advocacy groups (14) There are also efforts to normalize and depathologize the phenomenon, the attraction to objects is defined as a variant of orientation, a post-structuralist author - by analogy with heteronormativity - directly discusses “humanonormativity” (15). Psychological and psychiatric literature has explored the biological underpinnings of objectophilia, examining its relationship to autism, “personifying synesthesia,” and cross-modal mental imagery (3). While mechanophilic individuals aim to achieve sexual arousal and orgasm through interaction with vehicles, objectophiles’ attachment to various objects (walls, buttons, furniture, televisions, radios, computers, bridges, etc.) often lacks any sexual component. Objectophiles are predominantly women, whereas mechanophiles—like most individuals with paraphilias—are typically men (14, 15).

Limitations: The poor cooperation of the patient during assessment, the lack of direct observation of some behaviors, and the presence of multiple comorbid conditions (substance use, subsequent psychotic episodes) should be interpreted as limitations to the etiological inferences about the paraphilia and its relationship to other psychopathologies.

In this study, we presented a rare variant of fetishism—mechanophilia—through a case from forensic psychiatric practice, emphasizing the importance of thorough psychopathological and psychological assessment and differential diagnosis.

## Data Availability

The original contributions presented in the study are included in the article/supplementary material. Further inquiries can be directed to the corresponding author.
